# Optimization of electron microscopy for human brains with long-term fixation and fixed-frozen sections

**DOI:** 10.1186/2051-5960-2-42

**Published:** 2014-04-11

**Authors:** Xiao-Bo Liu, Cynthia M Schumann

**Affiliations:** 1 University of California, Davis, School of Medicine, Department of Pathology and Laboratory Medicine; Electron Microscopy Laboratory, Sacramento, USA; 2 University of California, Davis, School of Medicine, Department of Psychiatry and Behavioral Sciences, MIND Institute, Sacramento, USA; 3 UC Davis MIND Institute, 2805 20th Street, Sacramento, CA 95817, USA

## Abstract

**Background:**

Abnormal connectivity across brain regions underlies many neurological disorders including multiple sclerosis, schizophrenia and autism, possibly due to atypical axonal organization within white matter. Attempts at investigating axonal organization on post-mortem human brains have been hindered by the availability of high-quality, morphologically preserved tissue, particularly for neurodevelopmental disorders such as autism. Brains are generally stored in a fixative for long periods of time (often greater than 10 years) and in many cases, already frozen and sectioned on a microtome for histology and immunohistochemistry. Here we present a method to assess the quality and quantity of axons from long-term fixed and frozen-sectioned human brain samples to demonstrate their use for electron microscopy (EM) measures of axonal ultrastructure.

**Results:**

Six samples were collected from white matter below the superior temporal cortex of three typically developing human brains and prepared for EM analyses. Five samples were stored in fixative for over 10 years, two of which were also flash frozen and sectioned on a freezing microtome, and one additional case was fixed for 3 years and sectioned on a freezing microtome. In all six samples, ultrastructural qualitative and quantitative analyses demonstrate that myelinated axons can be identified and counted on the EM images. Although axon density differed between brains, axonal ultrastructure and density was well preserved and did not differ within cases for fixed and frozen tissue. There was no significant difference between cases in axon myelin sheath thickness (g-ratio) or axon diameter; approximately 70% of axons were in the small (0.25 μm) to medium (0.75 μm) range. Axon diameter and g-ratio were positively correlated, indicating that larger axons may have thinner myelin sheaths.

**Conclusion:**

The current study demonstrates that long term formalin fixed and frozen-sectioned human brain tissue can be used for ultrastructural analyses. Axon integrity is well preserved and can be quantified using the methods presented here. The ability to carry out EM on frozen sections allows for investigation of axonal organization in conjunction with other cellular and histological methods, such as immunohistochemistry and stereology, within the same brain and even within the same frozen cut section.

## Introduction

White matter in the brain is comprised of axons that connect and convey information across regions. The majority of axons are myelinated and send signals over long distances [1,2]. Disruptions in the connectivity of these neural pathways may underlie many forms of psychiatric and neurodevelopmental disorders including multiple sclerosis, schizophrenia and autism [1,3-5]. One option for evaluating the integrity of neural pathways in the brain is to utilize magnetic resonance imaging (MRI) techniques such as diffusion tensor imaging (DTI). However, fine structural abnormalities associated with axons remain generally undetectable at the resolution of MRI and therefore require postmortem human brain tissue to investigate these uniquely human disorders at the cellular level with electron microscopy (EM).

A few recent studies have utilized postmortem human brain samples for EM analyses to detect abnormalities in axonal ultrastructure, including postmortem samples to evaluate white matter in autism [5], schizophrenia [6] and surgically-removed tissue from epilepsy patients [7]. However, the often small sample sizes and limited human brains available for studies of axonal organization significantly impede progress in our understanding of these neurological and psychiatric disorders. In reality, many human brains have been fixed in formalin for long periods of time (over 10 years) in suboptimal conditions, and/or frozen and sectioned for neuropathology or immunohistochemistry. Therefore assessing the fine structure of axons with EM can often be problematic or preclude quantitative analyses. In addition, brain samples for the study of pediatric and neurodevelopmental disorders are rare and must be shared amongst several investigators employing different approaches and methods.

New approaches to preserve ultrastructural quality of human brain white matter and evaluate long term fixed and frozen samples are highly necessary in order to obtain quantitative information about the structural integrity of axon pathways in diseased and normal human brains. The benefits of developing such protocols are two-fold: One, it would increase the quantity of available tissue to share by utilizing preexisting long-term fixed and/or fixed-frozen sectioned brains, and two, the ability to apply other analyses, such as immunohistochemistry and stereology, to adjacent EM sections in order to evaluate multiple neuropathological mechanisms within the same brain. Here we present a modified method [8] from non-human primate tissue studies for optimal preservation and quantitative EM analyses using human brain tissues with long term fixation and flash-frozen cut human brain sections. The protocols presented here will allow for many more high quality human brains to be available and usable for study of ultrastructural changes in the white matter of the human brain.

## Materials and methods

### Tissue procurement

Three human brains were acquired in 2001 from the Department of Pathology at the University of California, Davis School of Medicine. All three cases were typically developing males with no major neurological or psychiatric conditions affecting the brain; the cause of death in each case was myocardial infarction. Case 1: 61 year old, postmortem interval (PMI, from time of death to fixation) 36 hours. Case 2: 66 year old, PMI 16 hours. Case 3: 44 year old, PMI 26 hours. After removal of the brain from the skull, each brain was bisected into two hemispheres. The right hemisphere was immersed in 10% buffered formalin and stored at 4° celsius in our laboratory for three years. The formalin in each container was replaced with fresh 10% buffered formalin approximately every two years. After three years, the right hemisphere from each case was coronally sectioned into ~5 cm thick blocks and processed as follows (Table 1).

**Table 1:** Tissue preparation and processing methods for electron microscopic imaging

**Steps**	**Long-term formalin fixed human brains**	**Fixed-frozen sectioned human brains**
1	Postfixed with 4% paraformaldehyde +2.5% glutaraldehyde in 0.1M PB for 2 weeks at 4°C	Postfixed with 4% paraformaldehyde +2.5% glutaraldehyde in 0.1M PB for 14 hours at 4°C
2	Vibratome section at 80 μm, store in 0.1M PB	Washed and stored in 0.1M PB
3	Osmification in OsO_4_ 0.1M PB for 20 min and washed in 0.1M PB	
4	Dehydration in graded ethanol solutions and 100% acetone	
5	1:1 acetone/Araldite for 1 hour at room temperature and in pure Araldite overnight at 4°C	
6	Flat embedding on siliconized slides with Araldite	
7	Polymeriztion at 60°C for 48 hours in oven	
8	Dissection of white matter from embedded sections and glue to blank resin blocks	
9	Thin sectioning at 70-80 nm and stain grids with uranyl acetate and lead citrate solutions	
10	Examination of ultrathin sections on electron microscope, image processing and quantification	

### Case 1 and 2

**Figure 1:**
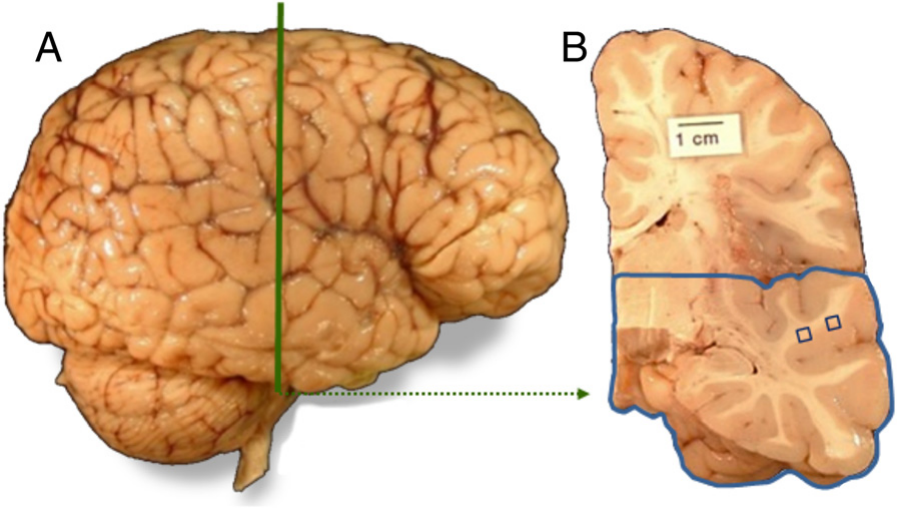
**Location of white matter sampling sites under STG. (A)** Lateral view of human brain showing the STG. Vertical line indicates location of coronal cross-section in (B). **(B)** Coronal brain slab through mid-rostrocaudal STG; squares indicate location of samples prepared for EM.

The tissue block containing the superior temporal cortex from each case at the level of the mid-rostocaudal hippocampus (Figure 1) was isolated and stored in 10% buffered formalin for an additional nine years at 4°C. An approximately 1 cm^3^ tissue block was then dissected from each to include the white matter dorsal (below) to the superior temporal cortex (Figure 1). Case 1 was further divided into two samples containing the medial (Case 1a) and lateral (Case 1b) portions to assess tissue quality within a single brain. Three tissue blocks (Case 1a, 1b, and Case 2) were photographed and transferred to 2% paraformaldehyde plus 2.5% glutaraldehyde in 0.1M phosphate buffer solution (4°C); then fixed for at least two weeks. After three rinses in cold 0.1M phosphate buffer, the blocks were further trimmed to approximately 1 cm × 1 cm × 0.5 cm in size. The blocks were cut with a vibratome (Leica) at 70-80 μm thickness; sections were collected and stored in cold 0.1M phosphate buffer (PB) in preparation for EM processing. An additional 1 cm^3^ tissue block was sampled from each case (1 and 2) and placed into a cryoprotectant solution (10% glycerol for two days and 20% glycerol) for five days in preparation for freezing. Each tissue block was flash frozen with 2-methyl butane (isopentane) and serially sectioned coronally into 50 μm thick sections. Two sections from each case were then washed in PB and transferred into 2% paraformaldehyde and 2.5% glutaraldehyde in 0.1M phosphate buffer solution (4°C) for 14 hours, then stored in cold 0.1M phosphate buffer in preparation for EM processing.

### Case 3

A 2 cm × 3 cm × 6 cm temporal lobe block from Case 3, after three years of storage in 10% buffered formalin at 4°C, was placed into a cryoprotectant solution (10% glycerol for two days and 20% glycerol) for five days in preparation for freezing. The tissue block was flash frozen with 2-methyl butane (isopentane) and serially sectioned coronally into six series of 50 μm thick sections and two series of 100 μm thick sections. One 100 μm series was stained with 0.25% thionin (standard Nissl method) for previously published stereological analyses [9]. One series of 50 μm thick sections was stored in tissue cryoprotectant solution (TCS) at -80°C for an additional nine years. From this series, three 50 μm frozen cut sections through the mid-rostrocaudal hippocampus were selected and washed in PB and transferred into 2% paraformaldehyde and 2.5% glutaraldehyde in 0.1M phosphate buffer solution (4°C) for 14 hours, then stored in cold 0.1M phosphate buffer in preparation for EM processing.

### Electron microscopic processing

Basic steps for EM processing are described in Table 1. For each case, approximately 4-5 sections were washed extensively in 0.1M phosphate buffer, transferred to 2% OsO_4_ in 0.1M PB for 20 min, and then washed three times in cold 0.1 M phosphate buffer. Sections were dehydrated in 70%, 90%, 95% and 100% ethanol and finally in 100% acetone for fifteen minutes. Sections were incubated in 1:1 Acetone/Araldite solution for one hour at room temperature, and transferred to pure Araldite for storage overnight at 4°C. Sections were embedded between silicon (Sigmacote, Sigma) coated coverslips and glass slides in freshly prepared Araldite and polymerized in 60–70°C oven for 48 hours. After polymerization, embedded sections were examined under a light microscope. The core of white matter approximately 2-3 mm below the gray-white matter boundary dorsal to the superior temporal gyrus, as shown in Figure 1, was identified and cut under a dissecting microscope; each piece of tissue was glued to a blank resin block. Each section was further trimmed and oriented on an ultramicrotome (Leica). We confirmed that the section contained the desired white matter by examining semi-thin sections (0.5 μm) stained by toluidine blue. Ultrathin sections at 70-80 nm were cut on the ultramicrotome and collected on Formvar coated single slot grids (Electron Microscopy Sciences). Grids were stained with uranyl acetate and lead citrate solutions, dried and stored in a grid box for EM imaging.

### Electron microscopic imaging

**Figure 2:**
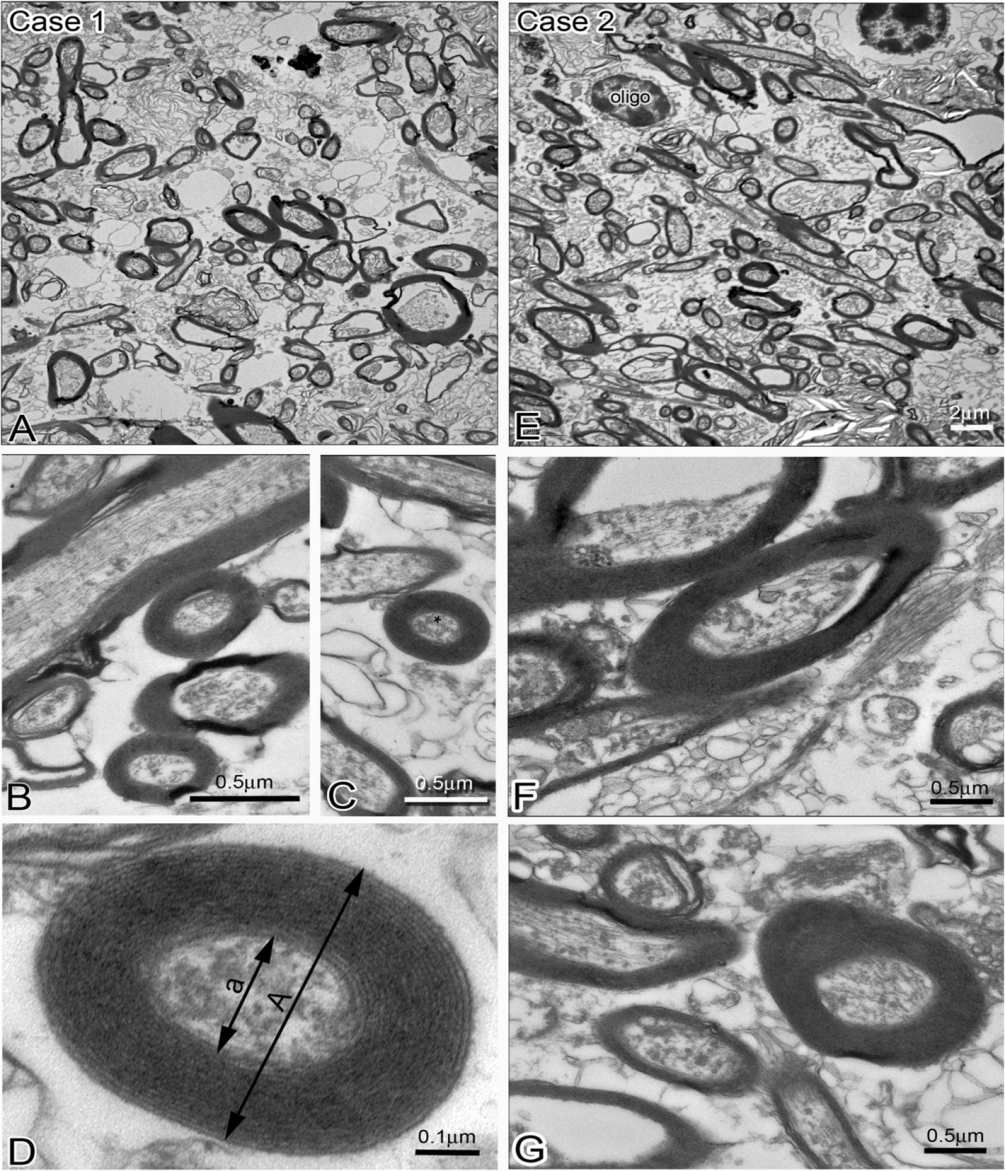
**Electron micrographs of myelinated axons in white matter below STG taken from two human brain samples case 1 and case 2. (A)**: Low magnification electron micrograph shows numerous myelinated axons of varying sizes in case 1. **(B, C)** High magnification EM demonstrates ultrastructural details of myelinated axons in cross and sagittal orientation. Note fine structure of microtubules and other subcellular organelles can be identified. The compactness of myelin sheaths differs among axons; bars = 0.5 μm. **(D)** Higher magnification EM of thick compact myelinated axon indicated in **(C)** by asterisk. Layers of myelin sheath can be clearly identified and counted. Axon diameter is defined by double arrow pointing to axoplasmic membranes labeled as **a**, the diameter of the myelinated fiber is defined by a double arrow pointed to the out layers of myelin sheath labeled as **A**; g-ratio = a/A. bar = 0.1 μm. **(E)** Low magnification EM shows myelinated axons and a few oligodendrocytes cell bodies in case 2. Note space between axon profiles is smaller compared to **(A)** indicating better structural preservation; bar = 2 μm. **(F, G)** High magnification EM showing some myelinated axons of varying sizes and myelin thickness. Note microtubules and neurofilaments can be clearly identified within axons; bar = 0.5 μm.

**Figure 3:**
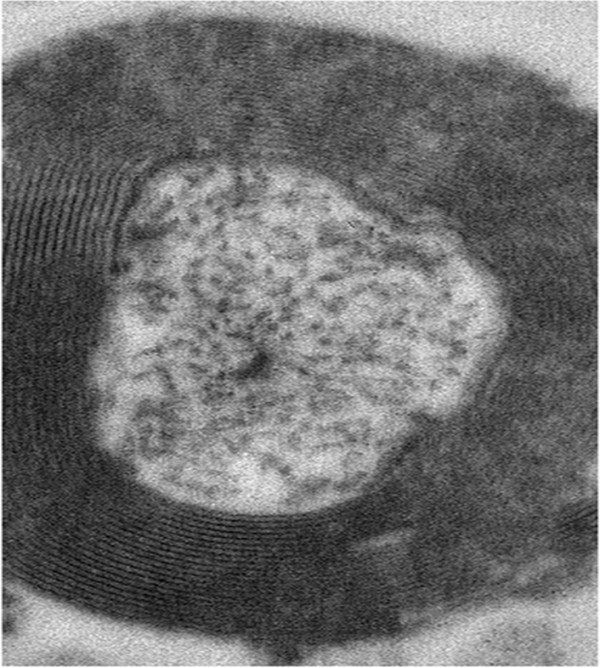
Electron micrographs of case 2 frozen sectioned samples at high magnification displaying myelinated axons with clear compact myelin sheaths and visible microtubules; bar = 200 nm.

**Figure 4:**
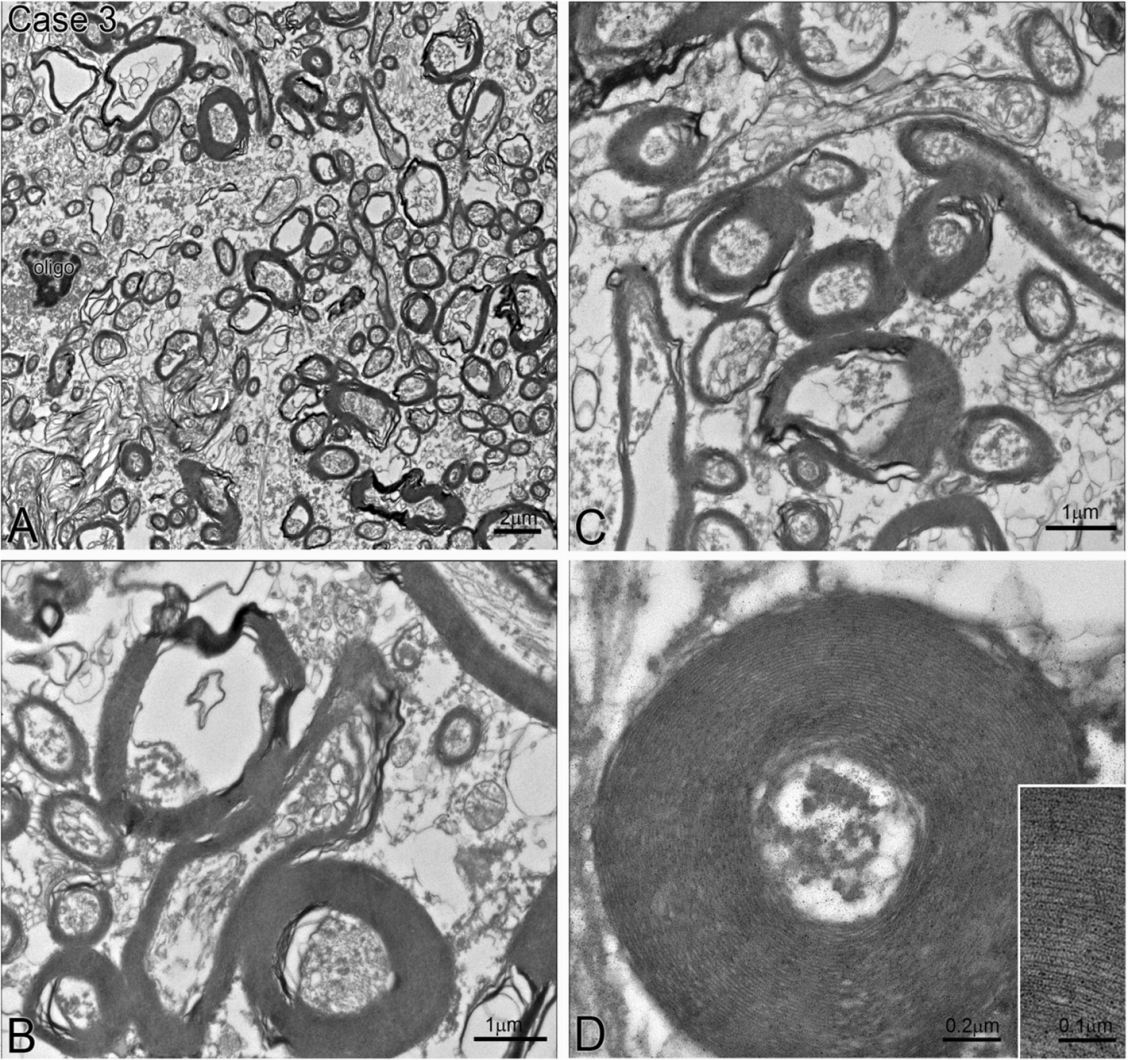
**Electron micrographs showing high white matter ultrastructural quality in frozen cut sections from case 3. (A)** Low magnification EM displaying numerous myelinated axons of varying sizes. Note an oligodendrocyte cell body located on left side of images; bar = 2 μm. **(B, C)** High magnification EM images showing myelinated axons with some subcellular organelles preserved, such as microtubules, neurofilaments and a few mitochondria; bars = 1 μm. **(D)** Higher magnification EM image showing a myelinated axon with numerous compact myelin sheaths. The inset demonstrates clarity of myelin sheath layers; bar = 0.2μm, bar in inset = 0.1 μm.

Grids were examined on a Philips CM120 Electron Microscope at 80 kV. Low magnification images of white matter regions containing axon bundles were taken at X4, 800 or X7, 000. To visualize the details of axons and other subcellular elements, additional images were taken at X15,000 or X20,000, so that the myelin sheaths and subcellular organelles such as mitochondria and neurofilaments could be clearly identified. From the EM samples examined, axons with myelin sheaths and axoplasmic organelles fit ultrastructural criteria for identification [10]. EM images were captured using a high resolution 2K X 2K CCD camera (Gatan, Pleasanton, CA) and imported into DigitalMicrograph (Gatan). To prepare Figures 2, 3, and 4, images were processed and imported to Adobe Photoshop (Adobe Systems, Mountain View) for further adjustments of brightness and contrast.

### Electron microscopic analysis and quantification

EM images were converted and saved as Tiff format to obtain quantitative measurements in Image J (NIH). We then carried out three types of analyses: (1) axon number density, (2) axon size, and (3) myelin sheath thickness (g-ratio), as described below.

### Axon density

This method was based on counting the number of myelinated axons in the defined unit area (imaging field of 560 μm^2^ at X7260), thus, the density of axons can be calculated as the number of axons per square micron.

### Axon size

Images were imported to Image J and the diameter of each axon was measured. Case 1(a, b) was pooled together for axon size analyses. We divided axons into groups based on diameter measures: small 0.25-0.5 μm; medium 0.5-0.8 μm; large >0.8 μm.

### Myelin sheath thickness

g-ratio calculation provides information about the thickness of the myelin sheath of individual axon fibers [11,12]. A high magnification image (X21,000) of myelinated axons was obtained and the image was imported to Image J for measuring the diameter of the axon (a, Figure 2D) and the myelinated axon caliber (A, Figure 2D). We measured at least 30 myelinated axons randomly chosen from each case. The ratio was calculated as the diameter of the axon (a) divided by diameter of the myelinated axon caliber (A): g-ratio = a/A. Thus, the smaller the g ratio, the thicker the myelin sheath layer. 

This study was exempt from Internal Review Board approval at UC Davis

## Results

### Qualitative observations of ultrastructural features in white matter

EM images (Figure 2A and E) at low magnification demonstrate the distribution pattern of myelinated axons in white matter of the superior temporal gyrus from fixed (never frozen) tissue. Surprisingly, axonal ultrastructure was well preserved and remarkably consistent between the fixed and fixed-frozen tissue samples collected from each of the cases 1 and 2. Compared to case 1, case 2 though exhibited considerably denser organization of myelinated axons (myelinated axons/μm^2^). The extraaxonal space was larger in case 1 samples than case 2, which also contained more identifiable structural contents (Figure 2F and 2G). Case 2 sample also preserved some cellular components, including oligodendrocyte cell bodies (Figure 2E). To visualize the structural details of myelin sheaths and quantify the myelin thickness, some myelinated axons were examined at higher magnification (× 21,000) (Figures 2D, 3 and 4D). In these images, myelinated axons from case 1 and 2 samples displayed similar ultrastructural features (Figure 2B, C, F and G), the myelin sheaths were in general very well preserved. The appearances of axoplasmic contents were variable, but in many images, microtubules and neurofilaments are clearly identifiable, whereas mitochondria were less easily identifiable. Axon ultrastructure appeared to be well preserved with numerous compact myelin sheaths, regardless of freezing, as showed in Figure 3 for case 2.

In case 3, fixed-frozen sectioned samples contained well-defined oligodendrocytes among myelinated axons, some subcellular organelles such as microtubules and neurofilaments, as well as mitochondria, could also be identified (Figure 4A-4C). At higher magnification, the layers of myelin sheath could be identified and counted. The axon diameter (a) and myelinated fiber diameter (A) were clearly identifiable and therefore could be quantified as described below.

### Quantitative analysis and comparison of different brain samples

#### ***Density of myelinated axons in the white matter***

**Figure 5:**
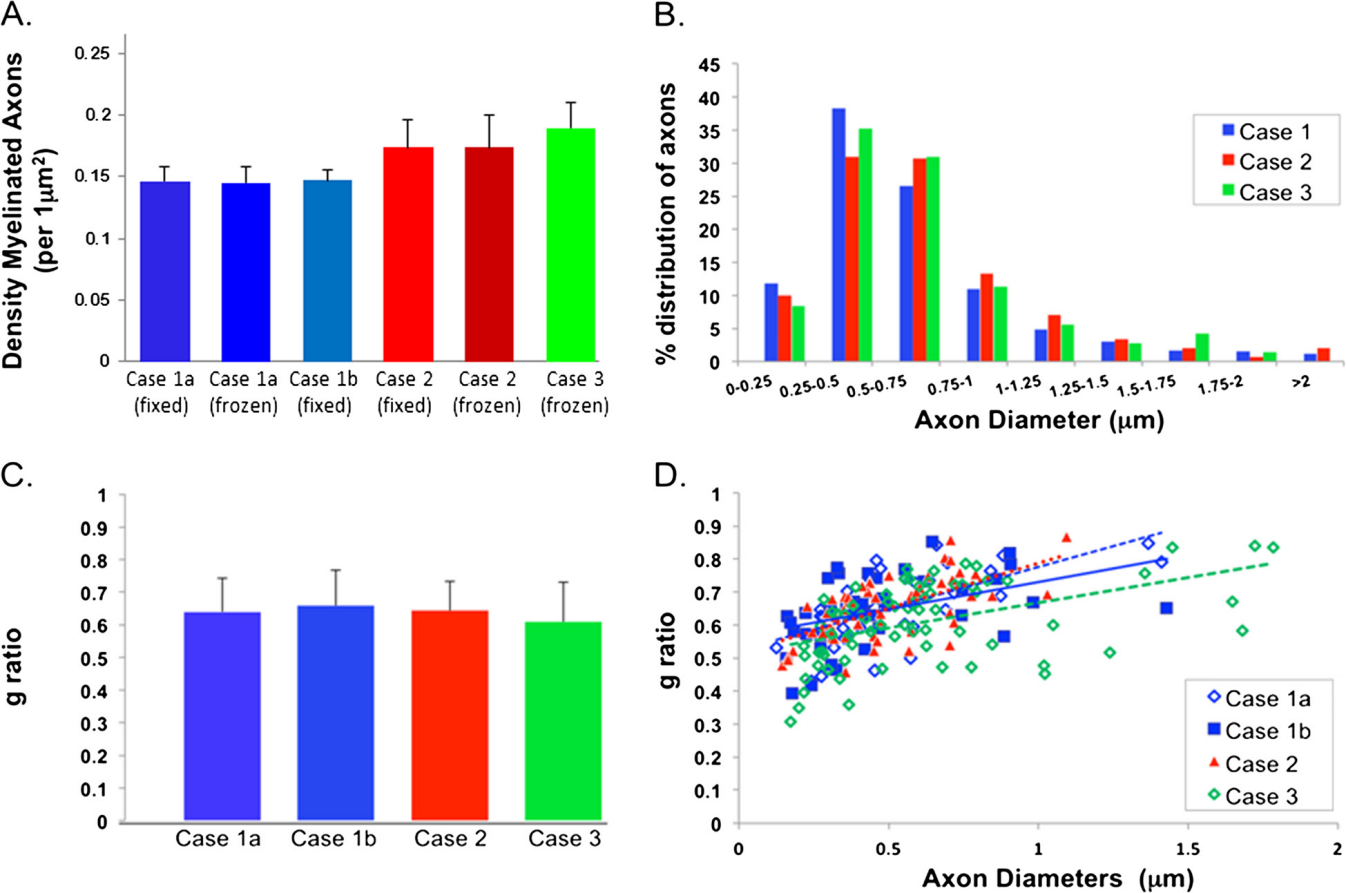
**Quantitative analysis of myelinated axons from EM samples of case 1a, b, 2 and 3. (A)** Histogram of myelinated axon density (mean number of myelinated axons per square micron (μm^2^). On average, 900 myelinated axons were counted in ~6000 μm^2^ area for each case. Case 1a fixed, case 1b fixed, and case 1 frozen do not differ in myelinated axon density. Case 2 fixed and frozen also do not differ in myelinated axon density. Case 2 and 3 have higher axon density relative to case 1 (p < 0.05), regardless of preparation method. **(B)** Histogram showing percentage distribution of myelinated axons size. Case 1a and case 1b are grouped as case 1. Graph displays similar size distribution pattern in case 1, 2 and 3. Approximately 70% of myelinated axons have diameters ranging from 0.25 μm to 0.75 μm. **(C)** g-ratio (average 0.63) histogram demonstrating no significant differences between cases. **(D)** Scatterplot showing positive correlation of g-ratio and myelinated axon diameter (r = .38, p < .01).

We counted the total number of myelinated axons in each EM image field (560 μm^2^, ×7260) to calculate mean myelinated axon density (per square micron region) for each sample (Figure 5A). There was a significant difference across all 6 samples in mean axon density (ANOVA, p < .001). As noted in our qualitative observations, we confirmed that all case 1 samples (mean = 0.14 ± 0.01 axons/μm^2^) are significantly less (p < 0.05), irrespective of freezing, than both case 2 and case 3 samples, which have densities 0.17 ± 0.02 axons/μm^2^ (case 2) and 0.19 ± 0.02 axons/μm^2^ (case 3) respectively. There is no significant difference (p =0.12) between case 2 and 3 samples. Within case 1, there was no difference in axon density between the two fixed samples (1a and 1b) and the frozen sample (ANOVA, p = .94). There was also no difference in axon density (mean = 0.17 ± 0.02 axons/μm^2^) between the fixed and frozen samples from case 2 (ANOVA, p = .98). These findings indicate that, although axon density differs between typically-developing brains, axonal ultrastructure is preserved through the freezing process presented here and does not impact measures of myelinated axon density.

#### ***Size distribution of myelinated axons in the white matter***

Axon diameter was measured in approximately 300 myelinated axons from each case. As shown in Figure 5B, we confirmed that the size distribution patterns of case 1, case 2 and case 3 are nearly identical irrespective of freezing preparation. Interestingly, in these samples, more than 70% of the axon diameters are in the range of 0.25 μm to 0.75 μm, indicating the majority of axons in the white matter under superior temporal cortex are in the small 0.25-0.5 μm to medium 0.5-0.8 μm size range. Mean axon diameters are as follows: Case1: 0.80 μm; case 2: 0.85 μm; case 3: 0.84 μm.

#### ***g-ratio analysis of myelin sheath thickness***

As shown in Figure 5C, mean g-ratio for each sample is as follows: case 1a: 0.64 ± 0.10; case 1b: 0.66 ± 0.11; case 2: 0.64 ± 0.09 and case 3: 0.61 ± 0.12. No significant difference was found in comparing all four samples (ANOVA, p = 0.7). There was a moderate difference between case 2 and 3 (p < 0.07). We then carried out a population analysis by examining g-ratio distributions and the correlation with axon diameters. As shown in Figure 5D, the g-ratio for each sample overlaps extensively for small to medium sized axons (0.25 to 0.75 μm in diameter); this result is consistent with the axon size distribution (Figure 5B). Interestingly, there is a positive correlation between g-ratio number and axon diameter (r = .38, p < .01), indicating that larger axons may have slightly thinner myelin sheaths.

## Discussion

In the present study, we sought to optimize methods for utilizing EM to examine the ultrastructural quality of white matter samples from long-term formalin fixed human brain tissue blocks and flash-frozen cut human brain sections. Using a modified EM protocol from previous animal studies [8,13-17], we analyzed three long-term fixed human brain samples and three fixed-frozen sectioned human brain sample. For the first time, this study demonstrates that white matter ultrastructure is well preserved and can be quantified in formalin fixed-frozen human brain sections, in addition to long term fixed human tissue sections, for studies of human brain neuropathology.

Qualitatively, we found that fixed-frozen sectioned tissue does maintain axonal ultrastructure in addition to well-defined oligodendrocytes, subcellular organelles, and clearly delineated layers of myelin sheath with the protocol presented here. There was no clear difference in axonal ultrastructure between tissue sections that had previously been frozen and those that had not. However, one of the two long term fixed brains (case 1: 61 year old, PMI 36 hours) displays an apparent decrease in myelinated axon density relative to the other long term fixed brain (case 2: 66 year old, PMI 16 hours) and to the fixed-frozen sectioned brain (case 3: 44 year old, PMI 26 hours), indicating that factors other than freezing may alter axon density, such as agonal state after death, PMI, variable storage time, or unknown brain pathology.

Quantitatively, confirming our qualitative observations, case 1 has significantly lower myelinated axon density relative to cases 2 and 3, irrespective of freezing. There is no significant difference in axon size distribution in all three brains, with diameters in the small to medium size range. The axon size range also confirmed the previous human frontal white matter study carried out by Zikopoulos and Barbas [5] with average axonal diameter of approximately 0.80-0.9 μm. These measures are also consistent with previous findings in other species; for example in fornix (0.81 μm), pyramid tract (0.9 μm) and optic tract (0.88 μm) of the guinea pig [18]. The thickness of the myelin sheath (as measured by g-ratio) also does not differ between the six samples. Surprisingly, our quantitative data also suggest that larger axons may have thinner myelin sheaths, however further study on a larger sample of cases would be required to confirm this finding.

From an evolutionary perspective, thicker myelinated axons may imply that longer connections and more reliable axonal conductions contribute significantly to the advancement of cognitive functions in higher mammals, including human. Interestingly, human brain g-ratio numbers differ from rodent brains [11], indicating that overall myelin sheath layers are thicker in human. The loss of myelinated axons during aging also reflects declining cognitive ability [19,20]. Moreover, loss of myelin layers in neurological diseases including multiple sclerosis and autism [5,21] strongly implicates that structural integrity of myelinated axons are crucial for maintaining normal neuronal function and more detailed analysis of normal and diseased brain tissues are highly required for future investigations.

### Applications for the study of human brain pathology

The need for EM analyses of human brain white matter has increased recently due to theories that many neurodevelopmental and psychiatric disorders have disruptions in neuronal connectivity. For example, DTI studies of schizophrenia have reported increased radial diffusivity that may reflect deficits in myelin integrity [22]. However, currently the low spatial resolution of DTI presents a considerable challenge of dissociating crossed fibers within a single voxel, and therefore limiting the detection of changes in axonal or myelin structure. A number of schizophrenia studies suggest abnormalities in the ultrastructure of myelin sheaths, reductions in oligodendrocyte numbers in some but not all brain regions, and dysregulation of myelin-associated gene expression, which could have profound effects on neuronal signaling [23-27]. One EM study found alterations in the ultrastructure of myelinated fibers and oligodendrocytes in prefrontal cortex [6]; however more studies are necessary in conjunction with immunohistochemistry [28] to characterize deficits in neuronal connectivity in patients with schizophrenia.

In autism spectrum disorder, numerous DTI studies support the theory of aberrant development of brain connectivity due to altered axonal microstructure [29]. In the only EM study of the autism brain published to date, Zikopoulos and Barbas [5] found evidence to support the hypothesis that myelinated axons of neurons in the prefrontal cortex may have increased local connectivity and decreased long-range connectivity. Specifically, they found significantly fewer large axons in the deep white matter below anterior cingulate cortex and a significantly greater density of smaller axons in superficial white matter of the same region. Unfortunately due to the lack of quality fixed tissue blocks available, this study was limited to a small sample size of five autism brains (one case also diagnosed with schizophrenia) and four control brains. Zikopoulos and Barbas [5], in addition to a large number of DTI studies [30], clearly demonstrate the need for larger scale studies of white matter ultrastructural pathology in multiple brain regions, given that ASD neuropathology is not limited to the prefrontal cortex [31]. From a technique perspective, the Zikopoulos and Barbas [5] study and the current study’s methods differed; in that they postfixed samples with microwave to enhance immunolabeling. In comparison to their approach, we preserved brain tissues in paraformaldehyde plus glutaraldehyde solution to omit microwaving procedures. In addition, their study was limited to fixed samples whereas the current study also demonstrated this method on frozen sections. With the method presented here, white matter ultrastructure was also well preserved, could be quantified, and combined with immunohistochemical and stereological studies within the same brain section. Given that high quality autism brain tissue, and in particular pediatric tissue, is likely more difficult to acquire than any other neurological or psychiatric disorder, novel methods such as the one presented here are necessary to shed light on the underlying neuropathological basis of aberrant brain connectivity in this disorder.

## Conclusions

**Figure 6:**
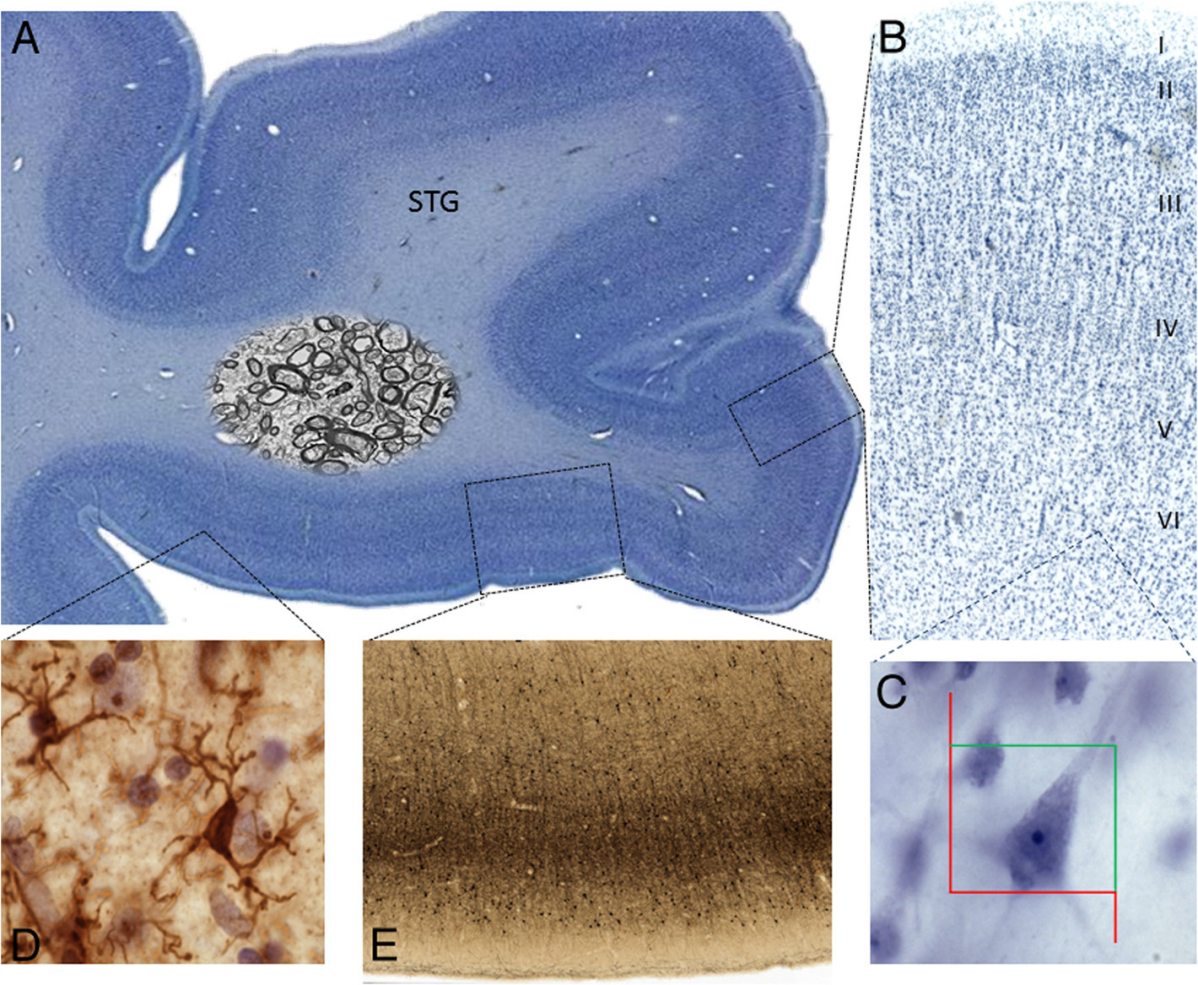
**Examples of potential preparations that can be applied to fixed-frozen sections adjacent to the STG section used for EM on case 3. (A)** Nissl (thionin) cell body stain. **(B)** Higher magnification of **(A)** showing layers of STG cortex. **(C)** Example of pyramidal neuron from **(B)** at 100× magnification used for stereological estimation of neuron number. **(D)** Iba-1 stained section for microglia. **(E)** Parvalbumin stained section through STG of case 3. *Additional protocols available upon request.*

The method presented here for preparing human brain long term fixed and fixed-frozen cut sections for EM opens an enormous opportunity for future large scale quantitative studies of axonal ultrastructure. Significantly, we can combine light microscopy for immunocytochemical or other histological labeling/stereological studies using the same serial sections (Figure 6 of case 3); thus a correlative neuropathological characterization can be carried out at the cellular and subcellular level. In addition, we can now use the same technique on human postmortem brains and non-human primate animal models of human disorders, such as autism and schizophrenia, to assess common features and the validity of the model.

A critical question is: *Can measures from varying preparations be combined?* In general, the preservation of white matter ultrastructure was consistent within and across cases. There was an approximately 15% reduction in axon density in case 1 relative to cases 2 and 3, however this finding appeared to be irrespective of freezing. Although the extra-axonal space varied from case to case, the axonal ultrastructure and axon size distribution were not compromised and are also comparable to results from previous studies on human brain samples [5,32]. Moreover, a similar g-ratio number indicates that myelin sheath thickness and integrity are not significantly altered due to long term fixation or sectioning method used. Therefore, although it is possible for measures of axon size distribution to be combined across methods, ideally, brain samples should be prepared with as consistent a protocol as possible. Taken together, this study demonstrates that long term formalin fixed and frozen cut brain sections can be used for EM study of ultrastructural analyses, however caution should be taken in interpreting the results of axon density and considerations made for other possible confounds when combining various preparation methods.

## Competing interest

The authors declare that the research was conducted in the absence of any commercial or financial relationships that could be construed as a potential conflict of interest.

## Author contributions

XL made a substantial contribution to the design of the study, acquisition and analysis of data, and drafted and revised the manuscript. CMS made a substantial contribution to the conception and design of the study, interpretation of data, and revised the manuscript. Both authors gave final approval of the version to be published and agreed to be accountable for all aspects of the work.

## References

[B1] Fields RD White matter in learning, cognition and psychiatric disorders Trends Neurosci 2008 2 361 370 10.1016/j.tins.2008.04.001 18538868 PMC2486416

[B2] LaMantia AS Rakic P Axon overproduction and elimination in the corpus callosum of the developing rhesus monkey J Neurosci 1990 2 2156 2175 2376772 10.1523/JNEUROSCI.10-07-02156.1990 PMC6570389

[B3] Makinodan M Rosen KM Ito S Corfas G A critical period for social experience-dependent oligodendrocyte maturation and myelination Science 2012 2 1357 1360 10.1126/science.1220845 22984073 PMC4165613

[B4] Nave KA Neuroscience: An ageing view of myelin repair Nature 2008 2 478 479 10.1038/455478a 18818646

[B5] Zikopoulos B Barbas H Changes in prefrontal axons may disrupt the network in autism J Neurosci 2010 2 14595 14609 10.1523/JNEUROSCI.2257-10.2010 21048117 PMC3073590

[B6] Uranova NA Vikhreva OV Rachmanova VI Orlovskaya DD Ultrastructural alterations of myelinated fibers and oligodendrocytes in the prefrontal cortex in schizophrenia: a postmortem morphometric study Schizophr Res Treatment 2011 2 325789 22937264 10.1155/2011/325789 PMC3420756

[B7] Concha L Livy DJ Beaulieu C Wheatley BM Gross DW In vivo diffusion tensor imaging and histopathology of the fimbria-fornix in temporal lobe epilepsy J Neurosci 2010 2 996 1002 10.1523/JNEUROSCI.1619-09.2010 20089908 PMC6633109

[B8] Freese JL Amaral DG The organization of projections from the amygdala to visual cortical areas TE and V1 in the macaque monkey J Comp Neurol 2005 2 295 317 10.1002/cne.20520 15846786

[B9] Schumann CM Amaral DG Stereological estimation of the number of neurons in the human amygdaloid complex J Comp Neurol 2005 2 320 329 10.1002/cne.20704 16175550 PMC2572713

[B10] Peters A Palay S Webster H The Fine Structure of the Nervous System 1991 New York: Oxford University Press

[B11] Chomiak T Hu B What is the optimal value of the g-ratio for myelinated fibers in the rat CNS? A theoretical approach PLoS One 2009 2 e7754 10.1371/journal.pone.0007754 19915661 PMC2771903

[B12] Marcus J Honigbaum S Shroff S Honke K Rosenbluth J Dupree JL Sulfatide is essential for the maintenance of CNS myelin and axon structure Glia 2006 2 372 381 10.1002/glia.20292 16288467

[B13] Freese JL Amaral DG Synaptic organization of projections from the amygdala to visual cortical areas TE and V1 in the macaque monkey J Comp Neurol 2006 2 655 667 10.1002/cne.20945 16615120 PMC2564872

[B14] Liu XB Honda CN Jones EG Distribution of four types of synapse on physiologically identified relay neurons in the ventral posterior thalamic nucleus of the cat J Comp Neurol 1995 2 69 91 10.1002/cne.903520106 7714240

[B15] Liu XB Warren RA Jones EG Synaptic distribution of afferents from reticular nucleus in ventroposterior nucleus of cat thalamus J Comp Neurol 1995 2 187 202 10.1002/cne.903520203 7721989

[B16] Needleman LA Liu XB El-Sabeawy F Jones EG McAllister AK MHC class I molecules are present both pre- and postsynaptically in the visual cortex during postnatal development and in adulthood Proc Natl Acad Sci U S A 2010 2 16999 17004 10.1073/pnas.1006087107 20837535 PMC2947898

[B17] Shen Y Liu XB Pleasure DE Deng W Axon-glia synapses are highly vulnerable to white matter injury in the developing brain J Neurosci Res 2012 2 105 121 10.1002/jnr.22722 21812016 PMC3209489

[B18] Perge JA Niven JE Mugnaini E Balasubramanian V Sterling P Why do axons differ in caliber? J Neurosci 2012 2 626 638 10.1523/JNEUROSCI.4254-11.2012 22238098 PMC3571697

[B19] Marner L Nyengaard JR Tang Y Pakkenberg B Marked loss of myelinated nerve fibers in the human brain with age J Comp Neurol 2003 2 144 152 10.1002/cne.10714 12794739

[B20] Zhang K Sejnowski TJ A universal scaling law between gray matter and white matter of cerebal cortex Proc Natl Acad Sci USA 2000 2 5621 5626 10.1073/pnas.090504197 10792049 PMC25878

[B21] Nave KA Myelination and support of axonal integrity by glia Nature 2010 2 244 252 10.1038/nature09614 21068833

[B22] Alba-Ferrara LM de Erausquin GA What does anisotropy measure? Insights from increased and decreased anisotropy in selective fiber tracts in schizophrenia. Front Integr Neurosci 2013 2 9 10.3389/fnint.2013.00009 PMC3593197 23483798

[B23] Fujino J Takahashi H Miyata J Sugihara G Kubota M Sasamoto A Fujiwara H Aso T Fukuyama H Murai T Impaired empathic abilities and reduced white matter integrity in schizophrenia Prog Neuropsychopharmacol Biol Psychiatry 2014 2 117 123 24099786 10.1016/j.pnpbp.2013.09.018

[B24] Hoistad M Heinsen H Wicinski B Schmitz C Hof PR Stereological assessment of the dorsal anterior cingulate cortex in schizophrenia: absence of changes in neuronal and glial densities Neuropathol Appl Neurobiol 2013 2 348 361 10.1111/j.1365-2990.2012.01296.x 22860626 PMC3508088

[B25] Lewis DA Cortical circuit dysfunction and cognitive deficits in schizophrenia–implications for preemptive interventions Eur J Neurosci 2012 2 1871 1878 10.1111/j.1460-9568.2012.08156.x 22708598 PMC3383640

[B26] Takahashi N Sakurai T Davis KL Buxbaum JD Linking oligodendrocyte and myelin dysfunction to neurocircuitry abnormalities in schizophrenia Prog Neurobiol 2011 2 13 24 10.1016/j.pneurobio.2010.09.004 20950668 PMC3622281

[B27] Voineskos AN Foussias G Lerch J Felsky D Remington G Rajji TK Lobaugh N Pollock BG Mulsant BH Neuroimaging evidence for the deficit subtype of schizophrenia JAMA Psychiatry 2013 2 472 480 10.1001/jamapsychiatry.2013.786 23467781

[B28] Glausier JR Fish KN Lewis DA Altered parvalbumin basket cell inputs in the dorsolateral prefrontal cortex of schizophrenia subjects Mol Psychiatry 2014 2 140 10.1038/mp.2013.177 PMC3874728 24217255

[B29] McFadden K Minshew NJ Evidence for dysregulation of axonal growth and guidance in the etiology of ASD Front Hum Neurosci 2013 2 671 24155705 10.3389/fnhum.2013.00671 PMC3804918

[B30] Zikopoulos B Barbas H Altered neural connectivity in excitatory and inhibitory cortical circuits in autism Front Hum Neurosci 2013 2 609 24098278 10.3389/fnhum.2013.00609 PMC3784686

[B31] Schumann CM Nordahl CW Bridging the gap between MRI and postmortem research in autism Brain Res 2011 2 175 186 20869352 10.1016/j.brainres.2010.09.061 PMC3050078

[B32] Pakkenberg B Pelvig D Marner L Bundgaard MJ Gundersen HJ Nyengaard JR Regeur L Aging and the human neocortex Exp Gerontol 2003 2 95 99 10.1016/S0531-5565(02)00151-1 12543266

